# Nutrient analysis of three low-carbohydrate diets differing in carbohydrate content

**DOI:** 10.3389/fnut.2024.1449109

**Published:** 2024-08-30

**Authors:** Lani Banner, Beth H. Rice Bradley, Jonathan Clinthorne

**Affiliations:** ^1^Simply Good Foods USA, Inc., Denver, CO, United States; ^2^Department of Nutrition and Food Sciences, University of Vermont, Burlington, VT, United States

**Keywords:** micronutrients, low-carbohydrate diet, diet, nutrient adequacy, low-carbohydrate, ketogenic diet

## Abstract

**Introduction:**

Low-carbohydrate diets are increasing in popularity. Despite clinical evidence demonstrating their safety and efficacy, concerns regarding the nutrient adequacy of low-carbohydrate diets persist. The aims of this study were to assess the nutrient adequacy of three 7-day meal plans that delivered 20 (VLCD20), 40 (VLCD40), and 100 (LCD100) grams of net carbohydrate per day respectively.

**Methods:**

Nutrient analyses were conducted using USDA Food Data Central.

**Results:**

All three low-carbohydrate meal plans exceeded recommendations for vitamins A, C, D, E, K, thiamin, riboflavin, niacin, B6, folate and B12 in males and females 31–70 years and exceeded calcium recommendations for adults 31–50 years but remained below the Tolerable Upper Intake Level. VLCD40 and LCD100 met or exceeded fiber recommendations for females ages 31–70 years and were adequate for males 51–70 years. None of the meal plans contributed meaningful amounts of added sugar. The plans exceeded the Recommended Dietary Allowance for protein for adults ages 31–70 years of age but were within the Acceptable Macronutrient Distribution Range of 10–35% of energy. The plans slightly exceeded recommendations for saturated fat and sodium but were lower in these nutrients than the average American diet and had more favorable omega-6 to omega-3 and sodium to potassium ratios than is typical. All three meals plans met or exceeded the Estimated Average Requirement for micronutrients in females ages 31–50 years, the population group most likely to consume low-carbohydrate diets.

**Discussion:**

Well-constructed low-carbohydrate meal plans can be nutritionally adequate in adults.

## 1 Introduction

The popularity of low-carbohydrate diets has doubled among the general US population over the last decade (1), and in particular, among middle-aged females (2). Low-carbohydrate dietary patterns are defined as those that contain less than 130 grams, or 10–25% of energy, from carbohydrate per day and include very low-carbohydrate dietary patterns containing 20–50 grams, or less than 10% of energy, from carbohydrate per day (Table 1). Other low-carbohydrate diets focus on energy provided by net carbohydrates, though follow a similar pattern regarding amounts of allowed daily net grams (3). Net carbohydrate is a term used to describe the total non-fiber saccharides that are digestible in humans (4) and is determined by subtracting non-digestible carbohydrate from total carbohydrate as data indicate this is a relatively accurate way to capture the overall glycemic load of the diet (5). Low-carbohydrate diets have been demonstrated to be clinically effective for the treatment of epilepsy (6, 7) and prevalent diet-related chronic diseases such as type 2 diabetes (T2D) (8), polycystic ovarian syndrome (PCOS) (9), metabolic syndrome (10), and overweight and obesity (11). Researchers have suggested that since cardiometabolic diseases disproportionately affect people from historically marginalized racial, ethnic, socioeconomic, and cultural backgrounds (12). A well-constructed low-carbohydrate dietary pattern may be one way for the Dietary Guidelines for Americans (DGA) to help address health equity in the US (13). A barrier to including low-carbohydrate diets in dietary guidance, however, is the current Acceptable Macronutrient Distribution Range (AMDR) for total carbohydrate being set at 45–65% of total energy, with a Recommended Dietary Allowance (RDA) of 130 grams total carbohydrate per day across all age and sex groups, a value that was based on the average amount of glucose utilized daily by the brain (14–16). Whereas clinical data for the effectiveness of low-carbohydrate diets to improve weight status and lipid levels are robust (17), concerns have been raised over their impact on micronutrient status (18), fiber intake, and potential contribution of excess protein, sodium, and saturated fat (2) to the diet.

**Table 1:** Low carbohydrate dietary patterns^1^.

Diet description	Carbohydrate (g/d)	Energy
Very-low carbohydrate	20–50	<10%
Low carbohydrate	50–129	10–25%

^1^Based on a 2,000 kcal/d eucaloric diet.

The aims of this dietary analysis were to assess the macro-and micronutrient content of three 7-day meal plans (20 grams net-, 40 grams net-, and 100 grams net carbohydrate per day, respectively) developed in collaboration with a nutritionist and present them in the context of the Dietary Reference Intakes (DRI).

## 2 Methods

Three 7-day meal plans containing 20 grams net-, 40 grams net-, and 100 grams net carbohydrates per day representing the lower- (VLCD20) and upper (VLCD40) ends of a very low-carbohydrate/ketogenic diet, and the upper middle-range of a low-carbohydrate diet (LCD100), respectively were developed in collaboration with a nutritionist (Table 2). The non-isocaloric meal plans were developed to reflect ranges of carbohydrate and calorie intakes in order to determine a broad view of nutrient adequacy of the different forms of carbohydrate restriction. Foods were selected for meal plans with a specific focus on addressing nutrients of public health concern identified in the 2020 Dietary Guidelines for Americans and foods that fit within guidance generally found in clinical studies of low carbohydrate diets as well as in guidance from professional societies regarding foods that fit into low carbohydrate dietary patterns. Specifically, meal plans were designed to mimic various dietary patterns utilized in studies of ketogenic and low carbohydrate diets as well as those used in commercial low-carbohydrate diet plans (19–21)—in order to assess whether these approaches could feasibly deliver adequate macro-and micronutrients. Nutrient analyses were conducted using USDA Food Data Central, which includes five distinct data types that provide information on food and nutrient profiles including: Foundation Foods, Food and Nutrition Database for Dietary Studies 2019–2020 (FNDDS 2019–2020), National Nutrient Database for Standard Reference Legacy Release (SR Legacy), USDA Global Branded Food Products Database (Branded Foods), and Experimental Foods (22).

**Table 2:** Seven-day low-carbohydrate meal plans^1^.

		Meal plans	
Day	VLCD20	VLCD40	LCD100
	*Breakfast*		
1	Vegetable cheese stack: 1 small tomato, ½ avocado, 2 C spinach, 1 tsp. olive oil, 1 oz. Monterey jack cheese, with 3.5 oz. sardines canned in tomato sauce, drained	VLCD20 Day 1 + ⅓ medium baked sweet potato	¾ medium sweet potato, 3.5 oz. sardines canned in tomato sauce, drained, 1 small tomato, 2 C spinach, ½ C blueberries
2	2 eggs scrambled with ½ C each of mushrooms and zucchini, sauteed with 1 TBS olive oil, and 3 oz. smoked salmon	VLCD20 Day 2 + 15 cherry tomatoes	2 eggs scrambled with ½ C each of mushrooms and zucchini, sauteed with 1 TBS olive oil, 15 cherry tomatoes, 1 slice whole wheat toast with 1½ tsp. butter
3	Chocolate-cinnamon smoothie: 1 C unsweetened coconut milk, ½ oz. chocolate whey protein isolate, ¼ tsp. cinnamon, 1 tsp. cocoa powder, ½ packet sucralose, 1TBS heavy cream	VLCD20 Day 3 + ⅓ C cooked rolled oats	VLCD20 Day 3 + ¾ C cooked rolled oats
4	4 oz. smoked salmon, 1 medium tomato, and 2 oz. mozzarella cheese	VLCD20 Day 4 + ½ medium sweet potato	VLCD20 Day 4 + 1 slice whole wheat toast, 1½ tsp. butter
5	2 large eggs, 1 tsp. olive oil, ½ avocado, and ¼ C salsa with 2 C spinach sauteed in 1 TBS butter	2 large eggs, 1 tsp. olive oil, ½ avocado, and ¼ C salsa, 2 C spinach, ¼ C low sodium black beans, canned	¼ C chopped tomatoes, 1 ½ cloves garlic, 1 jalapeno, 1 TBS lime juice, ¼ C cilantro, ¼ C chopped tomatillos, 2 eggs, 1 TBS olive oil, 2 C spinach, 2 almond-flour tortillas
6	Vanilla spinach shake: 1½ C almond beverage, 1 C spinach, 1 tsp. vanilla extract, 1 oz. vanilla whey protein isolate, 2 TBS flax seeds, 2 TBS heavy cream	VLCD20 Day 6 + ¼ C blueberries	Vanilla spinach shake: 1½ C almond beverage, 1 C spinach, 1 tsp. vanilla extract, 1 oz. vanilla whey protein isolate +1 C blueberries
7	Flax waffles: 3 TBS flax seeds, ½ oz. vanilla protein powder, ¼ tsp. baking powder, 1 pinch nutmeg, 1 pinch salt, 1 egg, 1 ½ tsp. canola oil, ½ tsp. vanilla extract, 1 TBS coconut milk, 1 ½ tsp. sucralose base sweetener, with 1 TBS butter and 2 TBS sugar-free syrup	VLCD20 Day 7 + 1 oz. roasted cashews	Orange sour cream waffles with fresh blueberry sauce: 2 TBS soy flour, ¾ tsp. baking powder, ¾ tsp. butter, ½ large egg, 4 tsp. sour cream, ¾ tsp. sucralose, ¾ tsp. orange zest, ½ C blueberries, 2 TBS roasted cashews

	*Lunch*		
1	Chicken-portobello broilers: 1 tsp. olive oil, 1.3 oz. boneless chicken breast, 1½ tsp. fresh scallions, 2/3 large egg, 1 portobello mushroom, sprinkled with 2 tsp. shredded mozzarella cheese, with 2 C marinated kale: 1 TBS olive oil, salt and pepper, to taste, 2C kale, ½ C shredded cabbage, ½ TBS cider vinegar	VLCD20 Day 1 + 1 TBS roasted cashews	Chicken-portobello broilers: 1 tsp. olive oil, 1.3 oz. boneless chicken breast, 1½ tsp. fresh scallions, 2/3 large egg, 1 portobello mushroom, sprinkled with 2 tsp. shredded mozzarella cheese, + 1 C red bell pepper slices, 1 oz. roasted cashews, tabbouleh salad: 4 tsp. wheat, bulgur, dry, ⅓ plum tomato, 1/6 cucumber, 2 TBS fresh parsley, 1 TBS fresh mint, ½ large scallion, 2 tsp. lemon juice, 1 ½ tsp. olive oil
2	Grilled lamb patties made with 6 oz. ground lamb, 1 oz. feta cheese, 1½ tsp. mint, with 5 oz. asparagus, 1 tsp. olive oil, salt and pepper, to taste.	Grilled lamb patties made with 6 oz. ground lamb, 1 oz. feta cheese, 1½ tsp. mint, with 7 large spears asparagus and ¼ cup canned chickpeas	⅓ C canned chickpeas, 7 large asparagus spears, cauliflower salad with salmon: ¾ C cauliflower florets, 1 TBS chopped onion, 1 TBS olive oil, ½ clove garlic, 3¾ oz. canned tomatoes, 1 TBS cilantro, 1½ tsp. red wine vinegar, 4 oz. canned salmon, 3/8 tsp. chili powder, ¼ tsp. paprika, pinch cumin, pinch cayenne, and 1/8 tsp. salt.
3	4 oz. roasted pork tenderloin, 3 C spinach, ½ Roma tomato, 4 medium asparagus spears, 1 TBS chopped red onion 1 TBS sunflower seeds with 2 TBS Greek vinaigrette (1½ tsp. lemon juice, ¼ clove garlic, ¼ tsp. salt, 1 pinch black pepper, 1½ TBS olive oil, ¼ tsp. red wine vinegar, 1 pinch dried oregano).	VLCD20 Day 3 + 3 medium asparagus spears	VLCD20 Day 3 + 3 medium asparagus spears, 1 medium sweet potato
4	6 oz. grilled chicken breast, 1 C asparagus and tomato salad: 5 cherry tomatoes, 5 basil leaves, 1 TBS olive oil, 2 tsp. balsamic vinegar	VLCD20 Day 4 + 1½ C spinach	VLCD20 Day 4 + 1½ C spinach, ½ C canned chickpeas
5	4 oz. canned tuna, 2 TBS mayonnaise, ½ C snap peas, ¼ C red bell peppers, 1 medium tomato, 2 TBS sunflower seeds	Same as VLCD20 Day 5	Same as VLCD20 Day 5 + 1 slice whole wheat bread
6	Veggie and pork bowl: 4 oz. pork tenderloin, 2 medium scallions, 2 oz. crimini mushrooms, ½ garlic clove, ¼ serrano pepper, 2.5 oz. Bok choy, ½ roma tomato, 1/8 oz. cilantro, and 1/8 oz. ginger root, ½ chicken bouillon cube, 1 ½ tsp. tamari, 1 ½ teaspoon canola oil	VLCD20 Day 6 + ¼ C cooked quinoa	VLCD20 Day 6 + 1 oz. brown rice pasta, dry
7	4 oz. chicken breast with 2 TBS sunflower seeds, marinated kale: 2 C kale, ½ C shredded cabbage, 1 TBS olive oil, 1½ tsp. apple cider vinegar, pinch of salt	VLCD20 Day 7 + 10 cherry tomatoes	VLCD40 Day 7 + ½ C cooked quinoa,

	*Dinner*		
1	Baked Salmon with Bok Choy: 5 oz. salmon, 6 oz. Bok choy, made with 1½ tsp. olive oil, ¼ TBS butter, pinch each of salt, pepper, and lemon zest, with 3/4 ounce roasted red peppers pureed with ½ ounce salsa	VLCD20 Day 1 + ¼ C cooked quinoa	VLCD20 Day 1 + ½ C cooked brown rice
2	Creamy Italian chicken and kale: ½ clove garlic, ¼ Roma tomato, ½ oz. bell pepper, ¾ tsp. olive oil, 1 tsp. chopped onion, ¼ C chicken broth, 1 TBS heavy cream, ½ tsp. red wine vinegar, ½ tsp. Italian seasoning, 4 oz. chicken breast, 2 C kale, 1½ tsp. cream cheese, 1 TBS parmesan cheese, with a pinch each of salt and pepper, served with ½ C frozen cauliflower rice.	VLCD20 Day 2 + 1 C cauliflower rice	Creamy Italian chicken and kale: ½ clove garlic, ¼ Roma tomato, ½ oz. bell pepper, 3/4 tsp. olive oil, 1 tsp. chopped onion, ¼ C chicken broth, 1TBS heavy cream, ½ tsp. red wine vinegar, ½ tsp. Italian seasoning, 4 oz. chicken breast, 2 C kale, 1½ tsp. cream cheese, 1 TBS parmesan cheese, with a pinch each of salt and pepper, + ½ C cooked quinoa
3	Spicy tomato-jalapeño chicken breast: 6 oz. chicken breast, ½ jalapeno, 1½ tsp. capers, ½ tsp. oregano, 3.5 oz. canned tomatoes with green chilis, 1½ tsp. olive oil, with 3 oz. green beans sauteed in 1½ TBS butter, salt and pepper, to taste	Spicy tomato-jalapeño chicken breast: 6 oz. chicken breast, ½ jalapeno, 1½ tsp. capers, ½ tsp. oregano, 3.5 oz. canned tomatoes with green chilis, 1½ tsp. olive oil, with 3 oz. green beans sauteed in 1½ tsp. butter, salt and pepper, to taste, ½ medium sweet potato	Spicy tomato-jalapeño chicken breast: 6 oz. chicken breast, ½ jalapeno, 1½ tsp. capers, ½ tsp. oregano, 3.5 oz. canned tomatoes with green chilis, 1½ tsp. olive oil, with 3 oz. green beans sauteed in 1½ tsp. butter, salt and pepper, to taste+2 almond-flour tortillas
4	½ lb. mackerel fillets, ½ tsp. fresh garlic, 1/8 tsp. salt, 1/8 tsp. dried rosemary, 1 TBS mayonnaise, ¾ tsp. Dijon mustard, with ½ C broccoli, 3 C kale sauteed in 1 TBS butter	5 1/3 oz. tilapia, 1 C broccoli flowerets, ¼ cup coconut cream, ¼ tsp. roasted red chili paste, 1/3 tsp. ginger, ¾ tsp. fish sauce, ½ tsp. sucralose-based sweetener, ½ C sliced red bell pepper, ½ tsp. lime juice, ¼ C cooked lentils	VLCD40 Day 4 + 1 Naan round
5	Baked meatballs: 3 oz. ground turkey, ¼ large zucchini, ½ oz. goat chest, 1¼ garlic cloves, 1/2 large egg, 1 ½ TBS heavy cream, ¼ tsp. Italian seasoning and 1 C frozen broccoli, 1 ½ tsp. olive oil, salt and pepper to taste	VLCD20 Day 5 + 1½ C cauliflower rice	VLCD20 Day 5 + ½ C cooked brown rice
6	Salmon cakes with avocado tartar sauce: 1/8 medium red bell pepper, ½ medium celery stalk, 4 oz. salmon, ¼ tsp. stoneground mustard,1 ¼ TBS mayonnaise, ¾ tsp. capers, 1 TBS parsley, ¼ large egg, ½ teaspoon Old Bay seasoning, ¼ avocado, ¾ tsp. lemon juice, 1/8 tsp. fresh dill, with 4 C spinach, sauteed in 1 TBS butter	Salmon cakes with avocado tartar sauce, 1½ C green beans, sauteed in 1 TBS butter	VLCD40 Day 6 + 1 medium sweet potato
7	Taco salad: 1½ C romaine lettuce, ¼ lb. ground beef, ¾ tsp. chili powder, ¼ tsp. cumin, 1/8 tsp. xanthan gum, 1/8 tsp. onion powder, 1/8 tsp. garlic powder, 1 oz. jicama, 1 TBS salsa verde, 1/8 C shredded cheddar, 1 oz. Monterey jack cheese, 1 TBS sour cream, with ½ avocado,	Taco Salad + ¼ C black beans	Spicy turkey and chickpea chili: 6 oz. ground turkey, 1 ½ tsp. olive oil, ¼ C chickpeas, ½ eggplant, ½ oz. shallots, 1 garlic glove, 1 TBS tomato paste, 1½ tsp. chili powder, ¼ tsp. cinnamon, ½ oz. cilantro, salt and pepper, to taste

	*Snacks*		
1	Muffin: 1 ½ tsp. erythritol, 1 tsp. ground cinnamon, ½ tsp. baking powder, 1 egg, 2 tsp. unsalted butter, served with 2 TBS cream cheese, 1 Commercially available low-net carbohydrate bar	Muffin: 1 ½ tsp. erythritol, 1 tsp. ground cinnamon, ½ tsp. baking powder, 1 egg, 2 tsp. unsalted butter, served with 1 TBS cream cheese, 1 Commercially available low-net carbohydrate bar	Same as VLCD40 + ½ C grapes
2	1 Commercially available low-net carbohydrate ready-to-drink shake, ½ an avocado with 1tsp sesame seeds, ¼ C fresh chopped jicama, and ¼ teaspoon Tajin seasoning	Same as VLCD20	1 Commercially available low-net carbohydrate ready-to-drink shake, ½ C sweet cherries, 1 serving (17 chips) sweet potato chips
3	1 Commercially available low-net carbohydrate bar, ½ avocado with 2 oz. Monterey jack cheese	Same as VLCD20	½ C cucumber slices, 3 oz. smoked salmon, 2 TBS cream cheese, ¾ C grapes, 1 commercially available low-net carbohydrate bar
4	1 oz. Havarti cheese, ¼ C jicama, 6 radishes, muffin in a minute: 1 ½ tsp. erythritol, 1 tsp. ground cinnamon, ½ tsp. baking powder, 1 large egg, 2 tsp. unsalted butter	Same as VLCD20	Muffin: 1 ½ tsp. erythritol, 1 tsp. ground cinnamon, ½ tsp. baking powder, 1 egg, 2 tsp. unsalted butter, ½ C sweet cherries, 1 C red bell pepper slices, 10 cherry tomatoes
5	1 Commercially available low-net carbohydrate ready-to-drink shake, 1 oz. Havarti cheese, 5 large black olives	Same as VLCD20 + ⅓ C blueberries	1 Commercially available low-net carbohydrate ready-to-drink shake, ¾ C blueberries, 1 serving (17 chips) sweet potato chips
6	½ avocado, 1 string cheese, 5 large black olives, 1 Commercially available low-net carbohydrate bar	Same as VLCD20	2 TBS cashews, 1 string cheese, 5 large black olives, 1 Commercially available low-net carbohydrate bar
7	½ medium red bell pepper, 2 oz. smoked salmon, 1 TBS cream cheese, ½ C sliced cucumber, 14 large black olives	Same as VLCD20	Same as VLCD20 + ½ C grapes

^1C, cup; oz, ounce; lb, pound; tsp., teaspoon; TBS, tablespoon; VLCD20, very low-carbohydrate diet delivering 20 grams of net carbohydrate per day; VLCD40, very low-carbohydrate diet delivering 40 grams of net carbohydrate per day; LCD100, low-carbohydrate diet delivering 100 grams net carbohydrate per day.^

In addition to a descriptive nutrient analysis, the RDA, intake levels of essential nutrients judged by the Food and Nutrition Board of the National Academies of Sciences Engineering, and Medicine to be adequate to meet the known nutrient needs of essentially all healthy persons, and used by federal, state, and local agencies to plan nutritionally adequate diets for individuals (15), was used as the reference value by which nutrient adequacy of the meal plans was measured for males and females ages 31–50 and 51–70 years. The Estimated Averaged Requirement (EAR), which estimates the daily level of nutrient intake necessary to meet the requirements of 50% of healthy individuals, and is used to plan nutritionally adequate diets for populations (15) was used as another reference value for determining nutrient adequacy among females ages 31–50 years, because according to data from six appended NHANES from 2007 to 2018, this population group is most likely to be following a low carbohydrate dietary pattern (2). Fifty-seven percent of self-reported low-carbohydrate dieters are females aged 48.67 years (2).

## 3 Results

The energy and macronutrient analysis of the three 7-day meal plans indicated they were adequate in energy for females ages 31–70 years, with VLCD20, VLCD40, and LCD100 having 91, 94, and 100% of the RDA for energy, respectively, in this population group (Table 3). For older females, ages 51–70, for whom energy recommendations decrease, the very low-carbohydrate/ketogenic meal plans, VLCD20 and VLCD40, met the RDA for energy, but LCD100 exceeded the RDA for energy by 12% in this population group. In males, VLCD20, VLCD40, and LCD100 fell short of recommended energy for both age groups.

**Table 3:** Nutrient analysis of low-carbohydrate meal plans for assessing adequacy against recommended dietary allowance (RDA) in females and males ages 31–50 years and 51–70 years^1,2^.

				RDA			
	Meal plan weekly average			Population group			
Nutrient	VLCD20	VLCD40	LCD100	Females 31–50 years	Males 31–50 years	Females 51–70 years	Males 51–70 years
Energy (kcals)	1643.2	1687.7	1798.7	1800	2200	1600	2000
Carbohydrate (g)	48.5	71.8	127	130	130	130	130
Net carbohydrate (g)	20.8	38.9	93.8	NV	NV	NV	NV
Fiber (g)	22.2	27.5	29.2	25.2	30.8	22.4	28
Protein (g)	113.9	118	115.7	46	56	46	56
Total Fat (g)	114.8	108.9	99	20–35% kcal	20–35% kcal	20–35% kcal	20–35% kcal
Saturated fat (g)	37.5	35.9	28.3	10% kcal	10% kcal	10% kcal	10% kcal
PUFA (g)	20.0	18.8	19.4	NV	NV	NV	NV
MUFA (g)	46.1	44.1	39.7	NV	NV	NV	NV
Omega-6 (g)	9.31	9.7	11.4	NV	NV	NV	NV
Omega-3 (g)	6.4	5.5	4.4	NV	NV	NV	NV
Omega-6:Omega-3	1.45	1.8	2.6	NV	NV	NV	NV
Vitamin A-RAE (μg)	961	1122	1467.9	700	900	700	900
Vitamin C (mg)	132.8	170.7	194.7	75	90	75	90
Vitamin D (μg)	20.3	15.8	17.2	600 IU (15 μg)	600 IU (15 μg)	600 IU (15 μg)	600 IU (15 μg)
Vitamin E (mg)	16.7	16.1	18.3	15	15	15	15
Vitamin K (μg)	483.5	418.4	412.7	90	120	90	120
Thiamin (mg)	1.3	1.6	1.7	1.1	1.2	1.1	1.2
Riboflavin (mg)	1.7	1.7	1.8	1.1	1.3	1.1	1.3
Niacin (mg)	29.7	29.2	31.9	14	16	14	16
Vitamin B6 (mg)	2.8	3.0	3.4	1.3	1.3	1.5	1.7
Folate (μg DFE)	410	476.2	449.1	400	400	400	400
Vitamin B12 (μg)	9.8	7.3	7.1	2.4	2.4	2.4	2.4
Choline (mg)	492.3	522	510.7	425	550	425	550
Calcium (mg)	1029	1121.9	1009.9	1000	1000	1200	1200
Iron (mg)	13.3	15.6	17.6	18	8	8	8
Magnesium (mg)	325.6	362.2	410.5	400	420	320	420
Phosphorus (mg)	1530.9	1639.3	1733	700	700	700	700
Sodium (mg)	2448.4	2464.9	2586	2300	2300	2300	2300
Potassium (mg)	3071	3488	3864.2	4700	4700	4700	4700
Sodium:Potassium	0.58	0.71	0.67	NV	NV	NV	NV

^1^DRI, Dietary Reference Intake; NV, no value; RDA, Recommended Dietary Allowance, VLCD20, very low-carbohydrate diet delivering 20 grams of net carbohydrate per day; VLCD40, very low-carbohydrate diet delivering 40 grams of net carbohydrate per day; LCD100, low-carbohydrate diet delivering 100 grams net carbohydrate per day. ^2^Nutrient analyses were conducted using USDA Food Data Central.

VLCD 20, VLCD40, and LCD100 had 37, 55, and 98% of the RDA for dietary carbohydrate in males and females 31–70 years of age, with less than half a percent of energy from added sugars. Whereas the dietary fiber content of VLCD20 fell short of the RDA for females 31–50 years of age, VLCD40 and LCD100 exceeded the RDA for dietary fiber in this population group by 9 and 16%, respectively. In older females, ages 51–70 years, VLCD20 had adequate dietary fiber, and VLCD40 and LCD100 exceeded the RDA by more than 20%. The three meal plans fell short of the RDA for dietary fiber in males 31–50 years of age, but VLCD40 and LCD100 met the RDA for dietary fiber in older males ages 51–70 years.

The meal plans were higher in fat to account for the lower carbohydrate content, with VLCD20, VLCD40, and LCD100 having 63, 58, and 50% of energy as fat. Saturated fatty acid content of the meal plans was 21, 19, and 13% of total energy from VLCD20, VLCD40, and LCD100, respectively. The omega-6 to omega-3 ratio was 1.45, 1.8, and 2.6 in VLCD20, VLCD40, and LCD100, respectively. The meal plans were higher than the RDA, but remained within the AMDR, for protein.

All three low-carbohydrate meal plans exceeded the RDA for vitamins A, C, D, E, K, thiamin, riboflavin, niacin, B6, folate and B12 in both males and females ages 31–70 years of age but remained well below the Tolerable Upper Intake Level (UL), the maximum daily intake unlikely to cause adverse health effects, for these vitamins. Whereas the meal plans had between 90 and 95% of the RDA for choline in males ages 31–70 years, they exceeded the RDA for females in these population groups. Calcium recommendations were exceeded in all three diets for males and females ages 31–50 years. Calcium recommendations increase for older adults ages 51–70 years of age, and all three diets fell short of meeting the RDA in these population groups. Whereas LCD100 exceeded the RDA for iron in males and females 51–70 years of age and males 31–50 years of age, VLCD20 and VLCD40 fell short of the RDA for iron in females 31–50 years of age for whom the iron requirement is higher. LCD100 had adequate iron for females 31–50 years with 98% of the RDA. Sodium exceeded the RDA by 6, 7, and 12%, and fell short of the RDA for potassium by 35, 26, and 18%, for VLCD20, VLCD40, and LCD100, respectively. The sodium to potassium ratio, however, was below one for all three meals plans with VLCD20, VLCD40, and LCD100 having sodium to potassium ratios of 0.58, 0.71, and 0.67, respectively.

When compared against the EAR for females ages 31–50 years, the three low-carbohydrate meal plans exceeded the EAR for protein, vitamins A, C, D, E, thiamin, riboflavin, niacin, B6, folate, and B12, and calcium, iron, magnesium, and phosphorus, but were within the AMDR for protein and well below the UL for all nutrients measured (Table 4).

**Table 4:** Nutrient analysis of low-carbohydrate meal plans for comparison with estimated average requirement (EAR) for females ages 31–50 years^1,2^.

	Weekly meal plan average			
Nutrient	VLCD20	VLCD40	LCD100	EAR for Females 31–50 years
Carbohydrate (g)	48.5	71.8	127	100
Protein (g)	113.9	118	115.7	0.66 g/kg/d
Vitamin A-RAE (μg)	961	1122	1467.9	500
Vitamin C (mg)	132.8	170.7	194.7	60
Vitamin D (μg)	20.3	15.8	17.2	10
Vitamin E (mg)	16.7	16.1	18.3	12
Thiamin (mg)	1.3	1.6	1.7	0.9
Riboflavin (mg)	1.7	1.7	1.8	0.9
Niacin (mg)	29.7	29.2	31.9	11
Vitamin B6 (mg)	2.8	3.0	3.4	1.1
Folate (μg DFE)	410	476.2	449.1	320
Vitamin B12 (μg)	9.8	7.3	7.1	2.0
Calcium (mg)	1029	1121.9	1009.9	800
Iron (mg)	13.3	15.6	17.6	8.1
Magnesium (mg)	325.6	362.2	410.5	265
Phosphorus	1530.9	1639.3	1733	580

^1^EAR, Estimated Average Requirement; NV, no value; VLCD20, very low-carbohydrate diet delivering 20 grams of net carbohydrate per day; VLCD40, very low-carbohydrate diet delivering 40 grams of net carbohydrate per day; LCD100, low-carbohydrate diet delivering 100 grams net carbohydrate per day. ^2^Nutrient analyses were conducted using USDA Food Data Central.

## 4 Discussion

This nutrient analysis of three low-carbohydrate meal plans designed in collaboration with a nutritionist aimed to assess nutrient adequacy in the context of the RDA for males and females ages 31–70 years and the EAR for those most likely to be following low carbohydrate diets, females 31–50 years (2). The results from this descriptive nutrient analysis demonstrated that when carefully constructed, low-carbohydrate meal plans intentionally designed to deliver less carbohydrate than the RDA can in fact deliver adequate fiber and micronutrients to the diets of Americans. The meal plans at the upper end of the very low-carbohydrate/ketogenic diet range that had 40 grams of net carbohydrate per day and the low-carbohydrate diet that had 100 grams of net carbohydrate per day met or exceeded fiber recommendations for middle-aged and older females and exceeded fiber recommendations for older males. This is important because dietary fiber consumption in the US is low enough to be of public health concern (23). Uniquely these meal plans contained commercially available foods low in net carbohydrates that were found in the USDA Global Branded Food Product Database, which ultimately provided on average 20, 16, and 15% of the daily fiber intake for VLCD20, VLCD40, and LCD100, respectively. While the meal plans did not meet the RDA for fiber in middle-aged males, this was in the context of diets that had less than 1,800 kcal per day. The dietary fiber RDA for males 31–50 years of age assumes a 2,200 kcal per day diet. When adjusted to meet the RDA for total calories in this population group, the low-carbohydrate meal plans can deliver adequate dietary fiber. Further, the low-carbohydrate meal plans did not contain meaningful amounts of added sugar. Current dietary guidance is to keep added sugars below 10% of total calories (23). The low-carbohydrate meal plans used in this analysis had less than half a percent of energy from added sugars.

Saturated fat was higher than recommended in the low-carbohydrate meal plans and is often a point of criticism in following such patterns. Since the introduction of the DGA nearly half a century ago, average American consumption of dietary saturated fat as a percent of total energy has been steadily declining while energy from dietary carbohydrate has been increasing (24). Despite which, the American population is metabolically unhealthy and living with diet-related chronic disease (23). Whereas the low-carbohydrate meal plans had higher saturated fat than recommended by the DGA, they also had a more favorable omega-6 to omega-3 ratio than that of the current American diet (25). Older American adults report consuming an omega-6 to omega-3 ratio of 7.8 on average and ranging between 3.1 and 20.3, however the low-carbohydrate meal plans in this nutrient analysis had an omega-6 to omega-3 ratio between 1.45 and 2.6. This is of importance, because a ratio of omega-6 to omega-3 fatty acids that is closer to 1 is associated with reduced risk of cancer, autoimmune-and inflammatory diseases, including cardiovascular disease (CVD) and diabetes (26). Another consideration when looking at the saturated fat content of the meal plans, is the food sources of saturated fat. It has been established that saturated fat in and of itself does not have a causal relationship with CVD (27) and that food sources of saturated fat have differential effects on biomarkers associated with coronary disease risk (28). Whole-milk fermented dairy foods such as cheese and yogurt have been associated with reduced risk of CVD in spite of their saturated fat content, perhaps in part because of their unique food matrix (29). Furthermore, it has been demonstrated that higher rates of fatty acid oxidation and reduced *de novo* lipogenesis occur on low-carbohydrate diets, which in turn decreases plasma concentrations of saturated fatty acids, indicating a reduced risk of cardiometabolic disease (30). Dietary fat metabolism is complex, and beyond the scope of this nutrient analysis, but the extent to which ketosis, the omega-6 to omega-3 ratio, and the foods used to deliver fatty acids to the diet factor into fat metabolism, and whether the saturated fat content of low carbohydrate diets is meaningful in this context, is worthy of more study.

The nutrient analysis of the low carbohydrate meal plans demonstrated that low-carbohydrate meal plans, including very low-carbohydrate/ketogenic plans, met and exceeded dietary recommendations for fat-and water-soluble vitamins for males and females ages 31–70 years, while remaining below the UL, dispelling concerns that low carbohydrate diets fall short of meeting vitamin recommendations. It is noteworthy that all low-carbohydrate meal plans exceeded recommendations for vitamin D, another nutrient for which consumption is low enough to be of public health concern (23). Regarding minerals, the low carbohydrate meal plans exceeded recommendations for calcium, another nutrient of public health concern (23), in males and females 31–50 years of age. Calcium recommendations are higher for older adults, and the low-carbohydrate meal plans fell short of calcium recommendations for males and females 51–70 years of age, indicating the need for such meal plans to be adapted for this population group. The low-carbohydrate meal plans exceeded sodium recommendations but fell short of potassium recommendations in males and females ages 31–70 years. This could be concerning considering Americans fall short on meeting potassium recommendations, making it another nutrient of public health concern, but overconsume sodium, which is associated with high blood pressure and increased risk of CVD (23). Notably, however, is that the sodium to potassium ratio of all three low carbohydrate meal plans was less than 1. According to data from the 2011–2012 NHANES, only 12.2% ± 1.5% of US adults had a sodium to potassium ratio that was less than 1 (28), and according to the World Health Organization, a sodium to potassium ratio of <0.49 is recommended to reduce risk of high blood pressure and CVD (31–33). The amount of potassium provided by the low-carbohydrate meal plans, albeit lower than amounts recommended by the DGA, was within the range that was observed to be protective against CVD events in the EPIC-Norfolk cohort (34).

When analyzed in context of the EAR for females 31–50 years of age, the low-carbohydrate meal plans exceeded the EAR for all vitamins and minerals for which an EAR exists, but remained below the UL, demonstrating that low carbohydrate dietary patterns can provide adequate micronutrients to the population most likely to consume them. Further, with the increasing popularity of low-carbohydrate diets among this population, there is perhaps a potential to close the gap on nutrient shortfalls, particularly for those nutrients for which consumption is low enough to be of public health concern, including calcium, vitamin D, potassium, choline, and dietary fiber (23). These results are in agreement with other micronutrient analyses that have been published previously (35–37).

The strengths of this nutrient analysis are that it assessed multiple days of low-carbohydrate meal plans on the lower and upper end of the very low-carbohydrate/ketogenic diet spectrum as well as at the upper end of what is considered a low-carbohydrate diet. Further it considered well-constructed low-carbohydrate meal plans in the context of the DRIs for individuals ages 31–70 years, but also for the female population group aged 31–50 years, who is currently most likely to be consuming a low carbohydrate diet. The meal plans were designed and analyzed using foods listed in USDA Food Data Central and can be replicated and modified to meet specific needs. Dietary patterns similar to the modeled LCD100 have also been proposed to be useful in place of calorie-restricted diets for people with type 2 diabetes in order to improve glycemic control, even in the absence of weight loss (38). To the best of our knowledge this is the first nutrient analysis of a 7-day meal plan for such non-ketogenic, low-carbohydrate diets.

The weaknesses of this analysis are that it did not utilize data from actual dietary intake among free-living subjects to construct meal plans from foods most commonly consumed and considered only nutrient adequacy of food without consideration of dietary supplement intake. Clinical trials that have compared the effectiveness and safety of various weight-loss diets have been limited by, and criticized for, short durations and low adherence (39). In a two-year trial in which 322 participants were randomly assigned to follow one of three weight-loss diets, and received no financial compensation for participation, 78% of participants assigned to the low-carbohydrate diet completed the study (39). The study design included regular support sessions with a dietitian. The importance of adequate education, resources, and support to successfully implement low-carbohydrate dietary patterns has been previously documented (40). The carbohydrate ranges utilized in the current analysis were modeled after various dietary patterns proposed in the low-carbohydrate diet literature (41). Whereas one cross-sectional study from Sweden found that in free-living subjects, low-carbohydrate dietary patterns could be sustained over time without apparent risk of deficiencies (42), investment in research to better understand the feasibility of long-term adherence to well-constructed low-carbohydrate dietary patterns is warranted at this time. As with any meal planning and modeling intended for population groups, however, findings cannot be individualized without consideration of age, gender, physical activity level, and preexisting conditions.

## 5 Conclusion

The results of this nutrient analysis indicate that well-constructed low-carbohydrate meal plans can be nutritionally adequate for adults, and in the context of a metabolically unhealthy population, the ratios of omega-6 to omega-3 fatty acids and sodium to potassium of low-carbohydrate meal plans should be taken into consideration.

## Data Availability

Publicly available datasets were analyzed in this study. This data can be found at: USDA FoodData Central https://fdc.nal.usda.gov/.
